# Pregnancy outcomes of elective induction in low-risk term pregnancies: A propensity-score analysis

**DOI:** 10.1097/MD.0000000000014284

**Published:** 2019-02-22

**Authors:** Eun Duc Na, Sung Woon Chang, Eun Hee Ahn, Sang Hee Jung, Young Ran Kim, Inkyung Jung, Hee Young Cho

**Affiliations:** a Department of Obstetrics and Gynecology, CHA Bundang Medical Center, CHA University, Seongnam; b Division of Biostatistics, Department of Biomedical Systems Informatics, Yonsei University College of Medicine, Seoul, Korea.

**Keywords:** cesarean delivery, elective induction of labor, pregnancy, propensity score analysis

## Abstract

We investigated the mode of delivery and perinatal outcomes in low-risk pregnant women whose labor was electively induced or expectantly managed at term.

Healthy women with viable, vertex singleton pregnancies at 37^+0^ to 40^+6^ weeks of gestation were included. Women electively induced (n = 416) in each week (37^+0^–37^+6^, 38^+0^–38^+6^, 39^+0^–39^+6^, 40^+0^–40^+6^ weeks) were compared with pregnant women with spontaneous labor (n = 487). The primary outcome was mode of delivery. A propensity score (PS) was derived using logistic regression to model the probability of elective induction group as a function of potential confounders. Altogether, 284 women with elective induction were matched with 284 women who underwent expectant management to create a PS-matched population. All analysis was performed using SAS software, version 9.4 (SAS Institute Inc., Cary, NC). All *P* values reported of the significance level was set at <.05.

There are no significant differences of delivery mode, neonatal intensive care unit (NICU) admission, and neonatal complication between PS-matched groups. Incidence of antepartum complications showed higher in the elective induction group compared to the spontaneous labor group (*P* = .04). When comparing each gestational week, incidence of NICU admission at 38 weeks in the elective induction group [10/74 (13.5%)] was significantly higher than in and the spontaneous labor group [2/74 (2.7%)] (*P* = .04).

Elective induction of labor at term is not associated with increased risk of cesarean delivery. However, overall incidence of NICU admission at 38 gestational weeks seems to be increased in elective induction.

## 1 Introduction

Induction of labor is a common obstetrical intervention with an incidence of 20% to 25% of pregnancies.^[1]^ There are medical indications for labor induction such as hypertensive diseases, maternal chronic diseases, fetal growth restriction, oligohydramnios, post-term pregnancy, and so on. Elective induction of labor is defined as an induction without any medical indications in healthy pregnant women and some experts suggested terminology of non-medically indicated inductions instead of elective induction.^[2,3]^ Elective induction could be considered with specific situations including geographical remoteness from the hospital, fatigue or discomfort associated with pregnancy, concerns that awaiting unexpectedly rapid labor and concerns about maternal or perinatal complications associated with continuing pregnancy.^[4]^ Elective induction of labor is a still debatable issue, and the relationship between elective induction of labor and the risk of cesarean delivery is not clear. It had been considered like a dogma that elective induction increased the risk of cesarean delivery.^[5,6]^ However, recent researches demonstrated unchanged or decreased risk of cesarean delivery and maternal and fetal morbidity with elective induction compared to expectant management.^[7,8]^ The reasons for these controversial results of existing literature could be different approaches of methods, different comparison groups, data sources, or gestational age. In Korea, in the absence of clear guidelines and recommendation of elective induction of labor, the use of elective induction varies according to the policy of each hospital.

The purpose of this study was to evaluate the benefits and harms of elective induction of labor in low-risk pregnant women using propensity score (PS) analysis.

## 2 Materials and methods

A total of 1,977 pregnant women whose delivery was carried out between January 2016, and November 2016, at Bundang CHA medical center, Korea were included and retrospectively analyzed. The approval was obtained from the Institutional Review Board of Bundang CHA medical center (CHAMC 2018–03–018).

Healthy pregnant women with viable, vertex singleton pregnancies at 37^+0^ to 41^+6^ weeks of gestation were included. We excluded pregnant women who delivered before 37 weeks or after 42 weeks, and women with prior cesarean section or previous uterine operation, multiple pregnancies, pregnancies with elective cesarean section and fetal anomalies were also excluded. There were 903 pregnant women who delivered between 37^+0^and 41^+6^ weeks of gestational ages, of which 416 patients underwent elective induction of labor and 487 were admitted with spontaneous labor or pre-labor rupture of membrane. Elective labor induction was conducted by pre-induction cervical ripening followed by oxytocin infusion in cases of unfavorable cervix or by oxytocin only in cases of favorable cervix. Bishop score was recorded at the time of admission.

We compared women who underwent elective labor induction and spontaneous labor or pre-labor rupture of membrane in each gestational week (37^+0^–37^+6^, 38^+0^–38^+6^, 39^+0^–39^+6^, 40^+0^–40^+6^ weeks). The primary outcome was mode of delivery, and the secondary outcomes were neonatal intensive care unit (NICU) admission, antepartum complications such as postpartum hemorrhage (PPH), vaginal wall hematoma, placental abruption and neonatal complications such as asphyxia, respiratory distress syndrome (RDS), bronchopulmonary dysplasia (BPD), necrotizing enterocolitis (NEC), and sepsis.

For statistical processing, the Chi-square test or Fisher exact test was used for categorical variables and the Two-sample *t* test or the Wilcoxon Rank Sum Test was used for continuous variables. A PS was derived using logistic regression to model the probability of elective induction group as a function of potential confounders. Altogether, 284 women with elective induction were matched with 284 women who underwent expectant management to create a propensity-score-matched population. All analysis was performed using SAS software, version 9.4 (SAS Institute, Inc., Cary, NC). All *P* value reported of the significance level was set at <.05.

## 3 Results

Total of 903 pregnant women was included in this study. After applying a PS-matching procedure, 284 patients who underwent elective labor induction were matched to 284 patients who had spontaneous labor. The PS-matching process further improved balance of the clinical characteristics between the elective labor induction and the spontaneous labor group (Table 1). Significant differences were observed between the elective induction group and the spontaneous labor group in terms of instrumental delivery (*P* <.001) and incidence of pregnancy-related complications (*P* <.041) in PS-matched population.

**Table 1:**
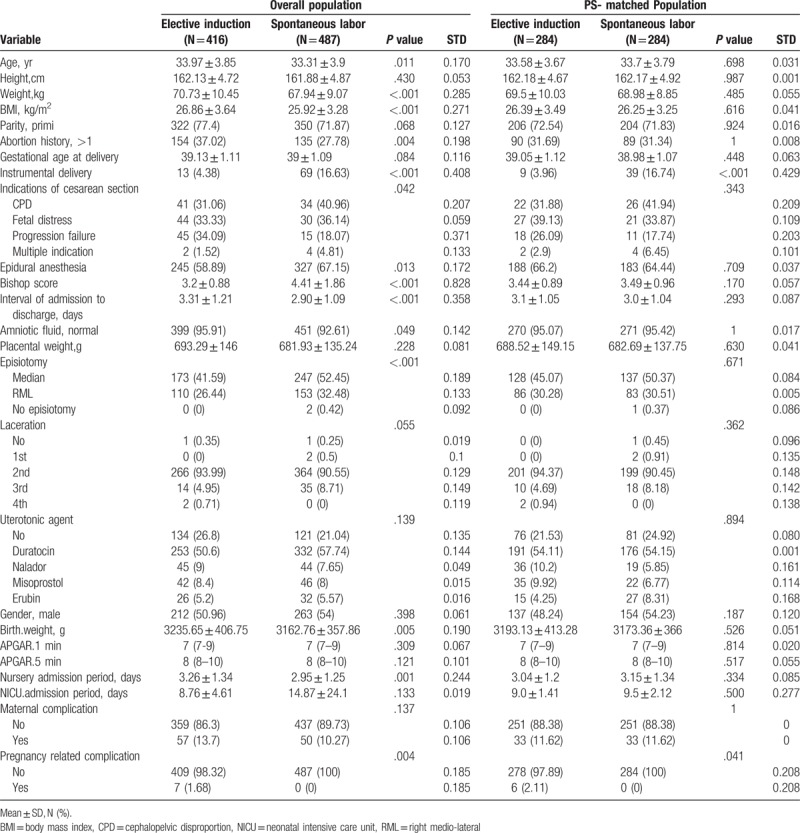
Clinical characteristics in the overall population and the PS-matched population.

Pregnancy outcomes between 2 groups were evaluated among the overall and PS-matched population (Table 2). There were statistically significant differences of delivery mode between the elective induction group and the spontaneous labor group in overall population (*P* <.001), but there were no significant differences of delivery mode in PS-matched population (*P* = .538). The incidence of NICU admission and neonatal complications was not significantly different among the overall and the PS-matched population. The incidence of antepartum complications was higher in elective the induction group compared to the spontaneous labor group (*P* = .041). When comparing each gestational week in the overall population, there was significant difference in the mode of delivery between the 39^+0^ to 39^+6^ group (*P* <.001) and the 40^+0^ plus group (*P* = .002, Table 3). However, we found no difference in the incidence of Cesarean section between 2 groups in the PS-matched population.

**Table 2:**
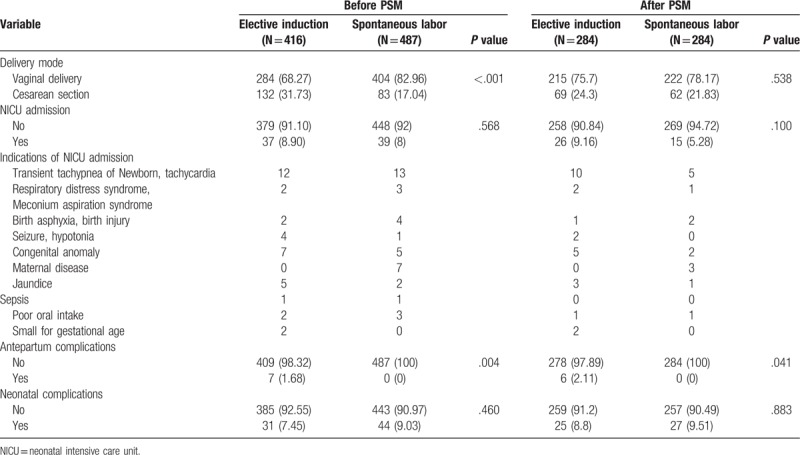
Pregnancy outcomes between study groups among the overall population and the PS-matched population.

**Table 3:**
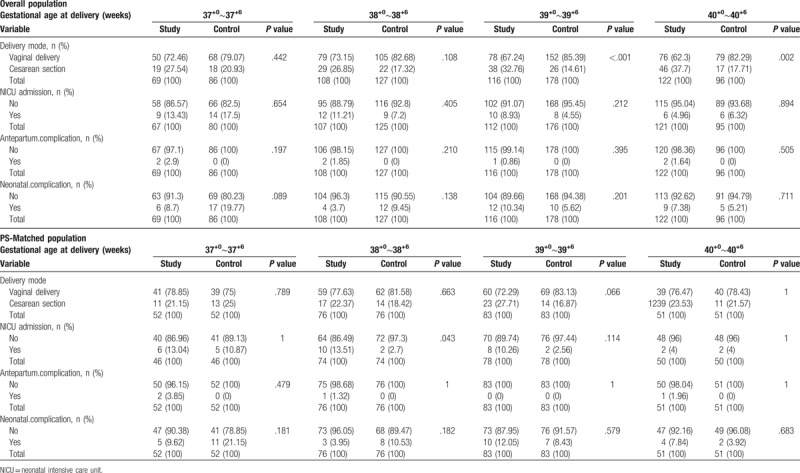
Comparison of clinical outcomes according to gestational age at delivery between the overall population and the PS-matched population.

The incidence of NICU admission was not different in the overall population according to gestational weeks, the incidence of NICU admission of the 38^+0^–38^+6^ group in the elective induction group [10/74 (13.5%)] were significantly higher than the spontaneous labor group [2/74 (2.7%)] (*P* = .043). There were no significant differences in antepartum and neonatal complications in 2 groups in the overall and the PS-matched population.

## 4 Discussion

This study compared maternal and perinatal outcomes between the elective induction group and the spontaneous labor group. We did not find significant differences of complications between 2 groups in the PS-matched population. This study showed that elective induction did not increase the risks of cesarean delivery, regardless of weeks of gestation, cervical status, and parity. Furthermore, the comparison of maternal and neonatal outcomes according to the gestational weeks from 37^+0^ to 40^+6^ didn’t show any statistically significant differences, except for the higher NICU admission rate among the gestational weeks from 38^+^ to 38^+6^ in the elective induction group.

There has been debate surrounding the safety of elective induction for long time. It is unsurprising that there were controversial conclusions in previous studies regarding the risk of adverse maternal and perinatal outcomes, including the risk of cesarean section.^[9,10]^ For decades, other studies have revealed that labor induction was associated with the increased risk of cesarean section, and cesarean delivery in the current pregnancy could affect to increase both maternal and neonatal complication in the next pregnancies.^[11]^ However, more recent randomized controlled trials (RCTs) have reported that cesarean delivery rate was not increased or decreased in the labor induction group compared to the expectant management group.^[12,13]^ A few studies have argued that elective induction can be used safely in special situations.^[14,15]^ Our finding of no increase of cesarean delivery rate with elective induction is consistent with the results of recent researches.

These discrepancies among studies can be attributed to the effect of flawed methodologies. Stock et al^[16]^ reported that there was no increased rate of cesarean delivery between pregnant women with elective labor induction and with expectant management, but they did not consider about bishop score of cervix nor analyze outcomes by parity. In 2009, previous prospective cohort study reported that cervix status and parity were important factors to affect the cesarean section rate with term pregnancies.^[17]^ Gibson et al^[18]^ evaluated the mode of delivery and maternal and neonatal outcomes in pregnant women with elective labor induction and expectant management at low-risk term pregnancies. Elective labor induction at term was associated with decreased rate of cesarean delivery and maternal and neonatal morbidities regardless of cervical status and parity.

Previous studies were mostly retrospective when they compared elective labor induction and expectant management.^[19]^ There were few RCTs, but there are not many well-designed or adequately powered studies.^[14,20,21]^ There can be many differences in the labor induction medication and protocols, and this can lead to controversial results according to the timing of the study or the institute. Our study, however, is a comparison of elective induction and spontaneous labor in a single institute, where standardization of labor management and consistent labor induction protocol is established, during 1-year period. A randomized clinical trial of a larger scale, using prospectively collected data from a well-characterized trial cohort, is ideal and necessary to validate the findings of this study but pregnancy is a sensitive situation that causes anxiety, and therefore, clinical trials maybe impractical. Our institute's management protocol uses as little external intervention as possible, thereby simulating a hypothetical randomized trial.^[22]^ Also, we used a PS-matched method to obtain the results that exclude confounding variables.^[23]^ All mentioned methodologies were used to minimize bias, therefore the results are more reliable even though this is a retrospective study.

Recent studies about elective induction have focused on the increase of cesarean delivery risk and maternal and neonatal outcome as the study outcome. However, previous study reported that labor induction group showed longer maternal hospital length of stay (10 h) than the expectant management group.^[24]^ In this study, interval of admission to discharge was significantly longer in the elective induction group than in the spontaneous labor group for the overall population. In addition, pregnant women expectantly managed would be visiting emergency clinic or labor and delivery room more frequently than those electively induced. Future researches should also consider the stress and anxiety of the obstetrician and the burden of work such as the length of hospital stay and the frequency of hospital. In addition, the potential risks of elective induction need to be weighed against the risks for dissatisfied experience of pregnant women and cost/resource use.

In conclusion, elective induction between 37^+0^ and 40^+6^ weeks of gestation is not strongly associated with an increased risk of Cesarean delivery and poor maternal and neonatal morbidity. However, incidence of NICU admission at 38 gestational weeks seems to be increased in elective induction in PS-matched population.

## Author contributions

**Conceptualization:** Hee Young Cho.

**Data curation:** Eun Duc Na, Hee Young Cho, Inkyung Jung.

**Formal analysis:** Eun Duc Na, Inkyung Jung.

**Investigation:** Eun Duc Na, Hee Young Cho.

**Writing – original draft:** Eun Duc Na, Hee Young Cho.

**Writing – review & editing:** Sung Woon Chang, Eun Hee Ahn, Sang Hee Jung, Young Ran Kim.

Hee Young Cho orcid: 0000-0001-7064-5056.
